# MMP2 and MMP9 serum levels are associated with favorable outcome in patients with inflammatory breast cancer treated with bevacizumab-based neoadjuvant chemotherapy in the BEVERLY-2 study

**DOI:** 10.18632/oncotarget.7612

**Published:** 2016-02-23

**Authors:** Emeline Tabouret, François Bertucci, Jean-Yves Pierga, Thierry Petit, Christelle Levy, Jean-Marc Ferrero, Mario Campone, Joseph Gligorov, Florence Lerebours, Henri Roché, Thomas Bachelot, Steven van Laere, Naoto T. Ueno, Yves Toiron, Pascal Finetti, Daniel Birnbaum, Jean-Paul Borg, Patrice Viens, Olivier Chinot, Anthony Gonçalves

**Affiliations:** ^1^ Institut Paoli-Calmettes, Medical Oncology Department, Marseille, France; ^2^ CRO2, Neuro-Oncology Department, UMRS_911, Marseille, France; ^3^ APHM, Marseille, France; ^4^ Centre de Recherche en Cancérologie de Marseille, Inserm, Marseille, France; ^5^ Institut Curie & Université Paris Descartes, Medical Oncology Department, Paris, France; ^6^ Centre Paul Strauss, Medical Oncology Department, Strasbourg, France; ^7^ Centre François Baclesse, Medical Oncology Department, Caen, France; ^8^ Centre Antoine Lacassagne, Medical Oncology Department, Nice, France; ^9^ Centre René Gauducheau, Medical Oncology Department, Saint-Herblin, France; ^10^ APHP-Tenon, APREC, Paris, France; ^11^ IUC-UPMC, Sorbonne University, Paris, France; ^12^ Hopital Rene Huguenin-Institut Curie, Medical Oncology Department, Saint-Cloud, France; ^13^ Institut Claudius Regaud, Medical Oncology Department, Toulouse, France; ^14^ Institut Leon Berard, Medical Oncology Department, Lyon, France; ^15^ Translational Cancer Research Unit, GZA Hospitals Sint-Augustinus, Wilrijk, Belgium; ^16^ Faculty of Medicine and Health Sciences, University of Antwerp, Antwerp, Belgium; ^17^ Department of Oncology, KU Leuven, Leuven, Belgium; ^18^ The University of Texas MD Anderson Cancer Center, Morgan Welch Inflammatory Breast Cancer Research Program and Clinic, Houston, TX, USA; ^19^ Centre de Recherche en Cancérologie de Marseille, CNRS, Marseille, France; ^20^ Aix-Marseille University, Marseille, France

**Keywords:** inflammatory breast cancer, bevacizumab, matrix metalloproteinase 2, matrix metalloproteinase 9, biomarker

## Abstract

**Purpose:**

Addition of bevacizumab to trastuzumab-based neoadjuvant chemotherapy in HER2-positive inflammatory breast cancer (IBC) was associated with favorable outcome in the BEVERLY-2 phase II trial. Circulating levels of matrix metalloproteinases (MMP) 2 and 9 were correlated to high response rate and prolonged survival in high-grade glioma treated with bevacizumab. We examined the prognostic impact of MMP2 and MMP9 serum levels in BEVERLY-2 patients.

**Experimental design:**

MMP2 and MMP9 serum levels were assessed using ELISA at baseline and before surgery in 45/52 available samples. Correlations were tested with pathological complete response (pCR), disease-free survival (DFS) and overall survival (OS).

**Results:**

Baseline (b) MMP2 and MMP9 serum levels were independent from patient characteristics and circulating tumor or endothelial cells, and were not correlated to pCR. High bMMP2 was correlated to better DFS (p=0.001) and OS (p=0.032), while low bMMP9 was correlated to better OS (p=0.022) and tended to be associated with longer DFS (p=0.071). In multivariate analyses, bMMP2 (p=0.003, Hazard Ratio [HR]: 0.115) and bMMP9 (p=0.041, HR: 3.511) remained correlated to DFS. As continuous variables, bMMP2 was associated with relapse (p=0.002) and death (p=0.049), while bMMP9 was associated with death (p=0.035). During treatment, significant increase in MMP2 and decrease in MMP9 levels (p<0.001 for both) were observed in 100% and 87% of patients respectively.

**Conclusions:**

High bMMP2 and low bMMP9 serum levels were associated with better survival in HER2-positive IBC patients treated with bevacizumab- and trastuzumab-based neoadjuvant chemotherapy. Their predictive value of bevacizumab benefit should be evaluated in a randomized trial.

## INTRODUCTION

Inflammatory breast cancer (IBC) is an uncommon (5% of all cases) but highly aggressive form of breast cancer [1]. IBC is clinically characterized by a rapidly growing edema and erythema of the breast involving at least 1/3 of the skin breast, and may include at the pathological level tumor emboli in the dermal lymphatics [2]. Even though significant improvements were achieved with multimodal treatment, the 5-year survival rate is only ∼ 40%, and the prognosis of IBC remains worse than that of non-inflammatory locally advanced breast cancer [1]. Biologically, IBC remains poorly understood but includes a high vascularity and an increased micro-vessel density consistent with a high expression of angiogenic factors, including the vascular endothelial growth factor (VEGFA) [2]. This led to the evaluation of anti-angiogenic therapies in this disease. In the recent open-label, single-arm, multicenter phase II BEVERLY-2 study, neoadjuvant and adjuvant bevacizumab was added to trastuzumab-based neoadjuvant chemotherapy and adjuvant trastuzumab in patients with nonmetastatic HER2-positive IBC leading to a high pathological complete response (pCR) rate of more than 60% [3] and promising 3-year disease-free survival (DFS) and overall survival (OS) rates of 68 and 90%, respectively. However, in the absence of a randomized design, the specific impact of bevacizumab was not established. In addition, the use of bevacizumab in breast cancer was recently challenged. Thus, biomarkers able to predict bevacizumab benefit in breast cancer, including HER2-positive IBC, would be of a crucial interest. Ideal biomarkers should be easily measurable, on multiple points upon treatment, and standardized [4]. Baseline levels and/or variation of numerous intratumor or circulating candidate prebiomarkers have been explored [5]. *In-situ* prebiomarkers such as VEGFA, VEGF-R2 or CA9, as well as circulating prebiomarkers such as VEGFA, VEGFR1, ICAM1, IL6, IL8 or circulating tumor cells count [6] were reported to predict bevacizumab benefit, but this predictive value was generally weak and rarely confirmed [5]. Matrix metalloproteinases 2 (MMP2) and 9 (MMP9) belong to the MMP family, whose activity is implicated in proteolysis of extra-cellular matrices, regulation of cell adhesion and migration, processing of growth factors and cytokines, and liberation of angiogenic factors [7]. We recently reported the association between the baseline circulating MMP2 level and, to a lesser extent, the MMP9 level, and the response rate, PFS and OS of patients treated with bevacizumab for recurrent high-grade glioma [8]. A baseline of high and low plasma levels of MMP2 and MMP9 respectively were associated with a high response rate and a prolonged progression-free survival and OS in recurrent high-grade gliomas treated with bevacizumab. Moreover, no association was found with patient survival in a similar population treated with cytotoxic agents only without anti-angiogenic therapy, suggesting a specific predictive value of these biomarkers.

BEVERLY-2 study included a prospective collection of serum samples before initiation of neoadjuvant chemotherapy and before surgical resection. In this study, our objective was to evaluate the prognostic impact of MMP2 and MMP9 serum levels, at baseline and during treatment, in patients with IBC treated with neoadjuvant bevacizumab, trastuzumab and chemotherapy.

## RESULTS

### Patient characteristics and baseline (b) MMP2 and MMP9 serum levels

Serums were available for 45 of 52 patients included in the trial. Characteristics of this patients' subset were similar to those of the entire population (Table 1). In our population, pCR was observed in 73.3% (95% Confidence Interval [CI]: 59.0-84.0) of patients. After a median follow-up of 3 years, 5 patients died and 14 presented disease recurrence. Median DFS and OS were not reached. The 3-year DFS and OS rates were 67.8% (95% CI: 55.2-83.3) and 88.1% (95%CI: 78.8-98.5), respectively.

**Table 1:** Patient characteristics of the 45 patients with available serum samples

Factors	N = 45	%
Age, median (range)	51,7 (37,0-67,9)	
Estrogen receptor-positive	16	35,6
Progesteron receptor-positive	7	15,6
Hormone receptors-positive	16	35,6
SBR grade		
2	18	41,9
3	25	58,1
Pathological response		
pCR	33	73,3
No pCR	12	26,7
Median follow-up (95%CI)	35.6 months	(35-36.2)
3-years DFS	73%	
3-years OS	91%	

CR: complete response; DFS: disease-free survival; OS: overall survival; 95%CI: confidence interval of 95%

At baseline, median bMMP2 and bMMP9 serum levels were 203.6 ng/ml (range, 116.2-338.9) and 629.6 ng/ml (range, 191-1189), respectively, and were inversely correlated (R= −0.498, p=0.001).

### bMMP2 and bMMP9 levels and correlation with histo-clinical features

We searched for correlations between the bMMP2 and bMMP9 serum levels and histo-clinical features. As shown in Supplementary Table 1, no significant correlation existed with patient's age, HR status and SBR grade. bMMP2 and bMMP9 levels were not associated with the count of baseline CTC and CEC, analyzed as continuous or qualitative variables.

### bMMP2 and bMMP9 serum levels and correlation with clinical outcome

**Figure 1:**
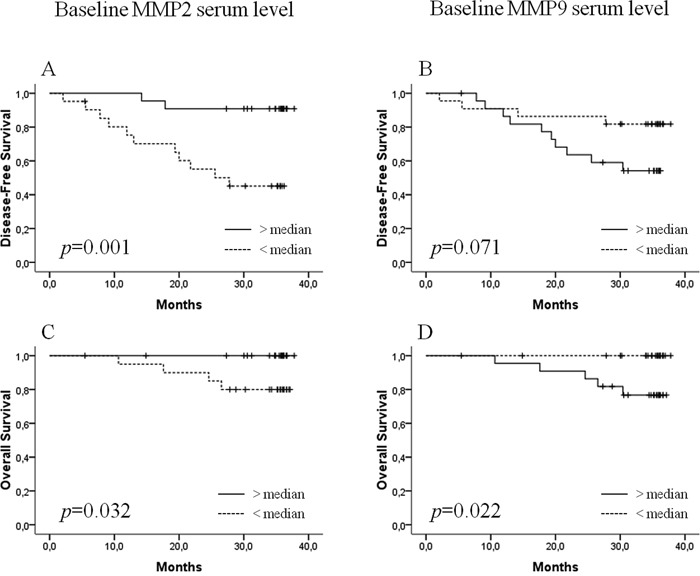
Disease-free survival (DFS) and overall survival (OS) according to baseline MMP2 **A, C.** and MMP9 **B, D.** serum levels.

Neither bMMP2 nor bMMP9 in continuous values were correlated to pCR (*p*=0.277 and *p*=0.104, respectively). So, for each MMP, subjects were divided into two groups (high and low bMMP) using the median value as cutoff. As binary variables, bMMP9 (*p*=0.208) and bMMP2 (*p*=0.146) were not correlated to pCR. By contrast, correlations existed with survival. In univariate analyses (Table 2), high bMMP2 was associated with longer DFS (*p*=0.001) and OS (*p*=0.032), whereas high bMMP9 was associated with worse OS (*p*=0.022) and tended to be associated with worse DFS (*p*=0.071) (Figure 1). Because of the low number of death events, multivariate analysis was only performed for DFS and included SBR grade (*p*=0.057). Both bMMP2 (*p*=0.003, HR: 0.115) and bMMP9 (*p*=0.041, HR: 3.511) remained significantly and oppositely correlated to DFS. Thus, 3-year DFS rates were 52.4% *versus* 91% for patients with low *versus* high bMMP2 serum level respectively, while DFS rates were 86.4% *versus* 56.5% for patients with low and high bMMP9.

**Table 2:** Univariate and multivariate (DFS only) analyses of MMP2 and MMP9 serum level at baseline and other potential prognostic factors

	DFS		OS
	Univariate	Multivariate*	Univariate^#^
**MMP2** *(cutoff: median)*	***0,001***	***0,006*** HR: 0.115 (0.025-0.533)	***0,032***
**MMP9** *(cutoff: median)*	***0,071***	***0,042*** HR: 3,511 (1,044-11,806)	***0,022***
HR-positive	*0,244*		*0,519*
ER-positive	*0,244*		*0,519*
PR-positive	*0,404*		*0,628*
pCR	*0,005*		*0,041*
SBR grade (2 versus 3)*	*0,057*		*0,418*
Age *(cutoff: median)*	*0,685*		*0,726*

* adjusted by SBR grade

# Multivariate analysis not allowed by the number of events.

**Figure 2:**
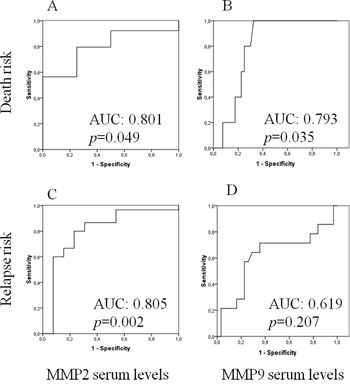
ROC analysis of the correlation between baseline MMP2 **A, C.** and MMP9 **B, D.** serum levels and the risks of death (A, B) and of recurrence (C, D).

As continuous variables, bMMP2 (*p*=0.021), but not bMMP9 (*p*=0.250) levels were correlated to DFS, whereas bMMP2 (*p*=0.098) and bMMP9 (*p*=0.068) levels presented a borderline significant trend for correlation to OS. Moreover, bMMP2 (*p*=0.049, AUC: 0.801) and bMMP9 (*p*=0.035, AUC: 0.793) levels were significantly correlated to the risk of death, while bMMP2 serum levels were correlated to the risk of recurrence (*p*=0.002, AUC: 0.805) (Figure 2).

### Exploratory scoring systems and ratio analysis

**Figure 3:**
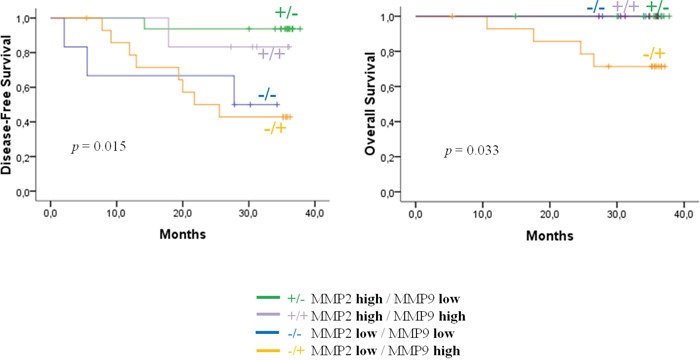
Disease-free survival and overall survival according to the four classes based on baseline MMP2 and MMP 9 serum levels Class 1: high MMP2 and low MMP9 (N=16), class 2: high MMP2 and high MMP9 (N=6), class 3: low MMP2 and low MMP9 (N=6) and class 4: low MMP2 and high MMP9 (N=15).

Given the opposite prognostic value of bMMP2 and bMMP9 levels, we divided our patient population in four classes: high bMMP2 and low bMMP9 (*N*=16), high bMMP2 and high bMMP9 (*N*=6), low bMMP2 and low bMMP9 (*N*=6), and low bMMP2 and high bMMP9 (*N*=15) serum levels. These four classes were significantly associated with DFS (*p*=0.015) and OS (*p*=0.033). All death events were observed in the worse subgroup defined by low bMMP2 and high bMMP9 serum levels (Figure 3). At the 3-year follow-up, DFS rates were 94% in the “high bMMP2/low bMMP9” class, 83% in the “high bMMP2/high bMMP9” class, 50% in the “low bMMP2/low bMMP9” class, and 43% in the “low bMMP2/high bMMP9” class, while OS rates were 100%, 100%, 100%, and 71%, respectively.

Finally, we evaluate the prognostic impact of the MMP9/MMP2 ratio, which was associated both with OS (p=0.044) and DFS (p=0.053).

### MMP2 and MMP9 serum levels changes during treatment and outcome

**Figure 4:**
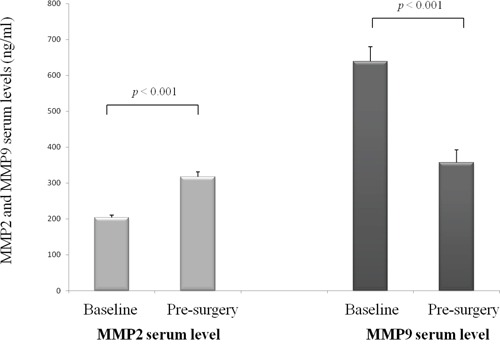
Changes of MMP2 and MMP9 serum levels under treatment, (mean, standard error of mean)

Post-neoadjuvant chemotherapy pre-surgery (ps) samples were available for 31 patients: median psMMP2 and psMMP9 serum levels were 298.5 ng/ml (range, 195.7-552.9) and 319.9 ng/ml (range, 20.1-858.4), respectively. Under treatment, all patients experienced an increase in psMMP2 serum level (*p*<0.001), while 87% of patients had a decrease in psMMP9 serum level (*p*<0.0.01) (Figure 4). psMMP2 and psMMP9 serum levels tended to be inversely correlated (R=−0.308, *p*=0.092). By contrast, the magnitudes of changes of MMP2 and MMP9 serum levels were not correlated (*p*=0.991) and were not associated with DFS or OS.

### Tumor MMP2 and MMP9 mRNA expressions in IBC

Since only a limited number of frozen tumor samples were available from BEVERLY-2 patients, we analyzed *MMP2* and *MMP9* mRNA expression in 137 clinical IBC samples profiled within the World IBC Consortium (E-MTAB-1006, E-MTAB-1547 and GSE22597). None of these patients received bevacizumab as part of their treatment. Their clinical characteristics are shown in Supplementary Table 2. Expression levels were heterogeneous across samples with a range of intensities over 3 decades in log10 scale (Figure S2). We searched for correlations (Fisher's exact test) between MMPs expression status (“high” *versus* “low” groups) and histo-clinical variables in IBC samples (Table S3). Regarding *MMP2* expression, no correlation was found with patients' age, histological type and grade, ER and PR, whereas high expression was associated with HER2-positive status (*p*=0.039). Regarding *MMP9* expression, no correlation was found with age, histological type and HER2 status, whereas high expression was associated with higher grade (*p*=0.020), ER-negative status (*p*=0.002) and PR-negative status (*p*=0.0007). Neither MMP2 nor MMP9 RNA expression levels were correlated to pathological response and to metastasis-free survival (Table S4).

## DISCUSSION

This analysis showed for the first time to our knowledge that, in the HER2-positive IBC patient population of the BEVERLY-2 trial, baseline MMP2 and MMP9 serum levels are associated with DFS and OS, are anti-correlated, and significantly changed during bevacizumab- and trastuzumab-based neo-adjuvant chemotherapy. These results are in accordance with the predictive value of these biomarkers for bevacizumab benefit, we recently reported in patients with recurrent high grade glioma [8]. Such predictive value was not found in glioma patients untreated with bevacizumab. Whether the same is true in HER2-positive IBC patients would ideally require a control group in a randomized design. To our knowledge, serum MMP2 and MMP9 dosages have not been reported in IBC, limiting access to some historical control. Despite that point, the similar results observed in glioma and HER2-positive IBC reinforce the interest of these biomarkers as predictive factors candidate for prediction of bevacizumab activity.

Given the increasing use and cost of antiangiogenic agents, and the heterogeneity of their benefit, particularly for bevacizumab, biomarkers of efficacy that could drive their therapeutic use, remain an unmet need in oncology [4]. In breast cancer, the use of bevacizumab was associated with a weak and debated activity, leading to the withdrawal of the FDA authorization and highlighting the especial need for predictive biomarkers. In the particular context of HER2-positive IBC, this is a critically important issue. Indeed, the BEVERLY-2 study demonstrated a high anti-tumor activity in terms of pCR and survival outcomes, comparing very favorably with historical controls [6]. However, other attractive strategies in this patient population may include optimization of anti-HER2 targeting using double blockade combination such as trastuzumab-pertuzumab [13] or trastuzumab-lapatinib associations [14]. Thus, predictive biomarkers that could help selecting among these two innovative approaches might be particularly useful.

Numerous studies explored potential predictive biomarkers for bevacizumab efficiency in breast cancer, but to date, none has been validated [5]. Hypertension and polymorphisms affecting the VEGFA pathway have shown some predictive value of bevacizumab benefit, although their limitations included lack of standardization, and inconsistent effect among studies. Tissue expressions of VEGFA, CD31, and PDGFRβ were associated with response, but their predictive value was not confirmed [15]. Moreover, baseline circulating biomarkers such as VEGFA, soluble VEGFR2, VCAM-1, and E-Selectin as well as circulating endothelial cells have been associated with outcome [16, 17]. However, their predictive value was inconsistent between studies. Recently, we reported that CTC count at baseline had an independent prognostic impact restricted to DFS in the BEVERLY-2 trial [6]. Interestingly, we did not find any correlation between baseline CTC count and MMP2 or MMP9 serum levels. We observed MMP serum levels variations during treatment: MMP2 increased for all patients and MMP9 decreased for most of them. Interestingly, we found similar results in our cohort of bevacizumab-treated glioma patients, with an increase of MMP2 and a decrease of MMP9 plasma levels during treatment before progression. In addition, in glioma patients, MMP2 plasma level significantly decreased at the time of progression. Variations of circulating markers were already reported under anti-angiogenic therapies, but their predictive values were limited [18]. Thus, MMP2 and MMP9 plasma levels could allow the initial patients' selection, but also could help in the treatment monitoring.

MMP2 and MMP9 belong to the matrix metalloproteinase (MMP) family, whose activity has been implicated in proteolysis of extra-cellular matrix. [7]. In breast cancer, MMP9 was reported to be highly expressed, in contrast to MMP2. Moreover, MMP2 seems to have no prognostic value for breast cancer patients while the prognostic impact of tissue expression or circulating level of MMP9 remains controversial [19, 20]. In this study, we have examined, for the first time in the literature, *MMP2* and *MMP9* mRNA tumor expression in a large population of IBC patients from the world IBC consortium but no relationship was found with pCR or survival. Of note, none of these patients had received bevacizumab in their initial neoadjuvant treatment. Thus, even though no data was available on corresponding serum levels, the lack of prognostic value observed for MMP2 and MMP9 tumor expression in this retrospective cohort may argue in favor of a predictive and specific impact of these markers for bevacizumab benefit.

One intriguing result of our study was the opposite correlation and prognostic impact of MMP2 and MMP9. MMP2 has been involved in angiogenesis, associated to pericyte recruitment, vascular maturation and functionality via multiple effectors [21, 22]. Interestingly, in orthotopic mouse model of glioblastoma completely devoid of MMP2 activity, tumor vascularity appeared to be less functional with reduction of VEGFR2 expression in tumor vessels and decrease of pericyte coverage [21]. In another model of lung cancer, MMP2 was reported to be implicated in the VEGFA expression by the tumor cells, through the PI3K/Akt pathway, underlining the major role played by MMP2 in the angiogenic process [22]. By contrast, MMP9 seems implicated in another vascularization process, called vasculogenesis, based on the recruitment of circulating endothelial and myeloid precursors [23]. Thus, these distinct roles in the tumor vascularization, supported by the inverse correlation of their serum concentrations, could explain their opposite predictive effect for bevacizumab activity.

Our study presented some limitations. The sample size of our population was small but related to the rarity of this disease. This limited number of patients and events could explain the borderline significance of our continuous analysis results. In this context, the exploratory results could be carefully interpreted taking into account these limitations.

However, the anti-correlation of the survival impact of these two markers evaluated in serum, their changes under bevacizumab as well as their similar impact observed in glioma treated with bevacizumab reinforce their potential value.

## MATERIALS AND METHODS

### Study design and patient population

BEVERLY-2 was a single-arm, multicenter, non-randomized phase II study. Study design, inclusion criteria and patient characteristics, as well as procedures, treatment (Figure S1, supplementary methods), efficacy and safety results have been previously published [3, 6]. All patients had a centrally reviewed HER-2 positive non metastatic IBC, defined as T4d (any N), or PEV2 or 3 according to the PEV (*Poussée EVolutive*) classification. Written informed consent was provided by patients before any screening procedure and was required for the translational research studies. The study was approved by the ethical board (Comité de Protection des Personnes Sud Méditerranée I) and registered (NCT00717405 and EUDRACT 2008-000783-16).

### Procedures and treatment

Patients received neoadjuvant chemotherapy consisting of 4 three-weekly cycles of FEC100 (500mg/m^2^ fluorouracil, 100 mg/m^2^ epirubicin, 500 mg/m^2^ cyclophosphamide) plus bevacizumab (15 mg/kg), followed by four 3-weekly cycles of docetaxel (100 mg/m^2^), bevacizumab (15 mg/kg), and trastuzumab (initially at a loading-dose of 8 mg/kg, and then a dose of 6 mg/kg). Surgical treatment consisted of mastectomy and axillary lymph node dissection. Bevacizumab was stopped 4 weeks prior to surgery and resumed (for a further 30 weeks) once the wound was healed entirely, during or after radiotherapy. Patients continued receiving trastuzumab (6 mg/kg) during the perioperative period which continued for another 30 weeks following surgery (42 weeks in total). Adjuvant radiotherapy was administered according to standard practice in combination with trastuzumab, and bevacizumab. Patients with hormone receptor (HR)-positive tumors received adjuvant hormone therapy.

### Blood sample collection and serum analysis

Additional blood samples (2 × 7.5 mL) were taken from patients at different times (supplementary methods): (1) before the first cycle of neoadjuvant chemotherapy and bevacizumab (baseline); (2) before the first trastuzumab administration during the neoadjuvant period (cycle 5); (3) before the surgical procedure (cycle 8); (4) during the adjuvant period before the reintroduction of bevacizumab (post-operative assessment); and (5) at the final visit at the end of adjuvant treatment (one-year follow-up). After collection of the whole blood, samples were allowed to clot at room temperature during 15-30 minutes. Then, serum was obtained by centrifuging at 2,000 x g for 10 minutes in a refrigerated centrifuge. Serum was immediately distributed into 0.5 ml aliquots, stored, and transported at −80°C or lower. Serums were thawed immediately before analysis. In the present study, serums at baseline and before surgery were analyzed for levels of MMP2 and MMP9 using commercially available enzyme-linked immunosorbent (ELISA) assay kits (R&D Systems^®^). Samples were run in duplicate, and the average was recorded. Serum levels were analyzed as continuous values and as binary values using the median value as cutoff. Circulating tumor cells (CTC) and circulating endothelial cells (CEC) counts were measured in blood samples as previously published [6] using CellSearch.

### mRNA expression of MMP2 and MMP9 in IBC tumor samples

MMP2 and MMP9 expressions have not been described in IBC tumor samples. Unfortunately, no frozen tumor sample from patients treated in the Beverly 2 trial was available for analysis. We thus analysed the gene expression dataset of the World IBC Consortium that included the whole-genome transcriptional profiles of 137 IBC profiled using Affymetrix DNA microarrays [9, 10]. The series and experiments have been described [9, 10], as well as the preprocessing of data. *MMP2* and *MMP9* mRNA expressions were measured by analyzing the 201069_at and 203936_at Affymetrix probe sets respectively, whose identity and specificity were verified using the NCBI program BLASTN 2.2.31+ and showed 100% accuracy. Expression was measured as discrete value by using the median expression level as cut-off: “high” expression was superior to the median, and “low” was inferior.

### Statistical analysis

For evaluation of pCR, Sataloff criteria were used [3]. Central review of tissue blocks was required for each patient and pCR was defined as a total or near total treatment effect with loss of nodal involvement (Sataloff classification TA and NA or NB) [11]. DFS was defined as time from first administration of neoadjuvant treatment to local, regional or distant recurrence, contralateral breast cancer, second primary cancer (other than squamous or basal cell carcinoma of the skin, melanoma *in situ*, carcinoma *in situ* of the cervix, colon carcinoma *in situ*, or lobular carcinoma *in situ* of the breast) or death from any cause. OS was defined using death from any cause. We analyzed MMP2 and MMP9 serum levels data with descriptive analyses, in terms of the variation between time points and correlation with pCR and survivals. Categorical variables were presented as frequencies and percentages, continuous variables as median and range. Comparisons of categorical variables distribution were performed by χ2 or Fisher's exact test. For continuous variables, non-parametric Mann-Whitney U test was used. The Kaplan-Meier method was used to estimate survival distributions. Log-rank tests were used for univariate comparisons. Cox proportional hazards models were used for multivariate analyses and to estimate hazard ratios in survival regression models. Multivariate analysis included all variables with a *p*-value < 0.10. Baseline and pre-surgical values of MMP2 and MMP9 serum levels were compared using with the Wilcoxon signed-rank test. Correlations were analyzed using the Spearman test. All reported *p*-values are two-sided, and *p*<0.05 was considered statistically significant. All statistical analyses were performed by paswstatistics.version21^®^. We followed the reporting REcommendations for tumor MARKer prognostic studies (REMARK criteria) [12].

## CONCLUSION

This analysis performed in the BEVERLY-2 phase II trial identified MMP2 and MMP9 as candidate biomarkers correlated consistently to DFS and OS in patients with HER2-positive inflammatory breast cancer. The prediction of bevacizumab benefit *versus* a prognostic value of MMP2/MMP9 should be assessed ideally in controlled studies. Further studies are needed to reinvestigate the biological role of MMP2 and MMP9 and their interaction with the VEGFA pathway.

## SUPPLEMENTARY FIGURES AND TABLES




