# Supplementary material for: MMP2 and MMP9 serum levels are associated with favorable outcome in patients with inflammatory breast cancer treated with bevacizumab-based neoadjuvant chemotherapy in the BEVERLY-2 study
Source: Oncotarget. 2016 Feb 23;7(14):18531–40. doi: 10.18632/oncotarget.7612 (PMC4951307; doi:10.18632/oncotarget.7612)
Supplement: Supplementary file 1 [file oncotarget-07-18531-s001.pdf]

SUPPLEMENTARY FIGURES AND TABLES

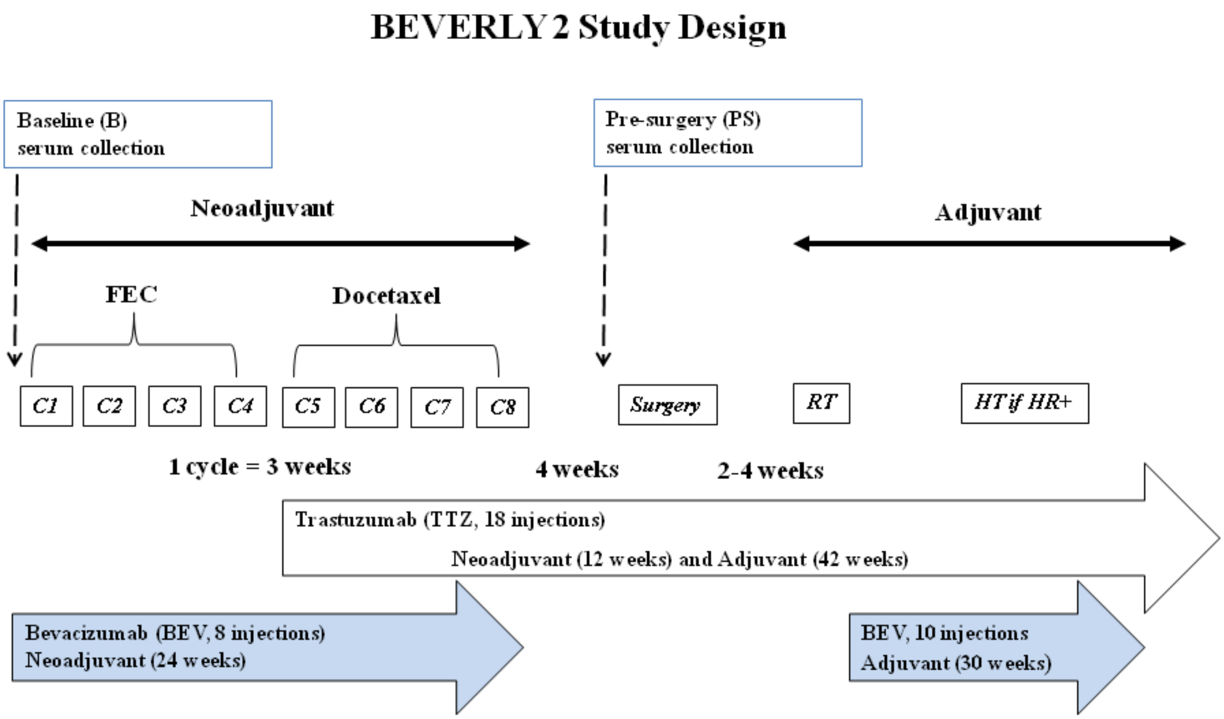

Supplementary Figure S1: Study design.

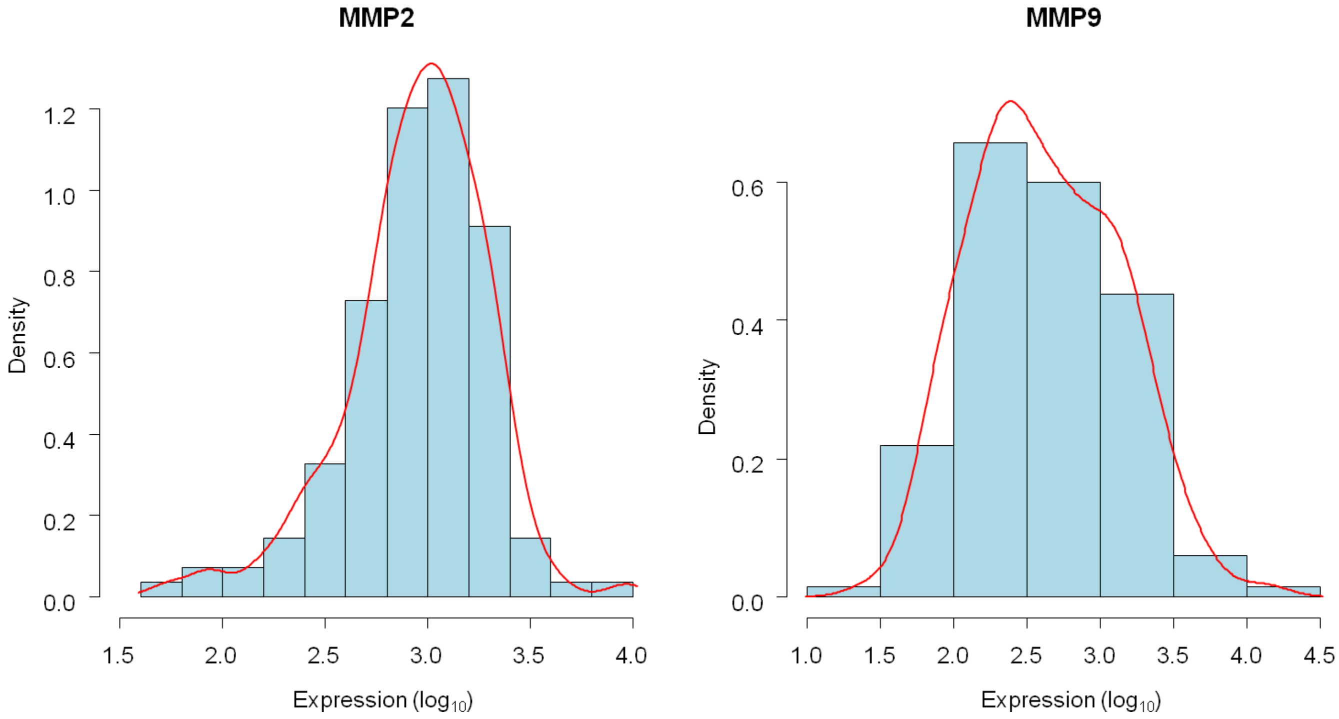

Supplementary Figure S2: Histogram of distribution of *MMP2* and *MMP9* expression levels across the 137 IBC samples (log10 scale) after normalization. The red line represents the density curve of distribution.

Supplementary Table S1: Correlation between MMP2 and MMP9 serum levels and patient characteristics

	Pre-CT (baseline)		Post-CT (presurgery)	
	**MMP2** ***p*** **value**	**MMP9** ***p*** **value**	**MMP2** ***p*** **value**	**MMP9** ***p*** **value**
**Age** (continuous)	0,679	0,133	0,743	0,213
**Hormone receptors**				
ER-positive	0,248	0,061	0,480	0,292
PR-positive	0,468	0,635	0,590	0,681
HR-positive	0,248	0,061	0,480	0,292
**SBR** grade (1&2 *vs* 3)	0,731	0,273	0,536	0,145
**CTC**				
Positive/Negative	0,276	0,371	*NA*	*NA*
Continuous	0,136	0,430	*NA*	*NA*
**CEC**				
Positive/Negative	0,531	0,070	*NA*	*NA*
Cutoff: median	*NA*	*NA*	0,265	0,571
Continuous	0,946	0,236	0,082	0,603

Supplementary Table S2: Histoclinical characteristics of 137 IBC samples (mRNA expression in pre-treatment tumor samples)

Characteristics*	N (%)
Age, average (min-max), years (135)	50 (24-82)
Histological type (135)	
ductal	122 (90%)
lobular	7 (5%)
mixt	3 (2%)
other	3 (2%)
Histological grade (131)	
1	0 (0%)
2	30 (23%)
3	101 (77%)
ER status (137)	
negative	66 (48%)
positive	71 (52%)
PR status (137)	
negative	88 (64%)
positive	49 (36%)
ERBB2 status (137)	
negative	95 (69%)
positive	42 (31%)
Pathological complete response (87)	
no	59 (68%)
yes	28 (32%)
5-year MFS** (121)	50% [95%CI 41-61]
Follow-up**, median (months)	58

*, the number of cases with available information is shown between commas
**, AJCC stage III patients only

Supplementary Table S3: Correlations of MMP2 and MMP9 tumor expression levels with histo-clinical characteristics in IBC

Characteristics	N	MMP2 groups		*p* value	MMP9 groups		*p* value
		MMP2-low	MMP2-high		MMP9-low	MMP9-high	
Age, average (min-max), years	135	48 (24-82)	54 (28-78)	0,036	51 (27-82)	49.5 (24-80)	0,573
Histological type				0,123			0,686
ductal	122	69 (95%)	53 (85%)		62 (87%)	60 (94%)	
lobular	7	2 (3%)	5 (8%)		5 (7%)	2 (3%)	
mixt	3	2 (3%)	1 (2%)		2 (3%)	1 (2%)	
other	3	0 (0%)	3 (5%)		2 (3%)	1 (2%)	
Histological grade				1,000			0,021
2	30	16 (23%)	14 (23%)		22 (31%)	8 (13%)	
3	101	55 (77%)	46 (77%)		48 (69%)	53 (87%)	
ER status				1,000			0,002
negative	66	36 (48%)	30 (48%)		25 (35%)	41 (62%)	
positive	71	39 (52%)	32 (52%)		46 (65%)	25 (38%)	
PR status				0,722			0,001
negative	88	47 (63%)	41 (66%)		36 (51%)	52 (79%)	
positive	49	28 (37%)	21 (34%)		35 (49%)	14 (21%)	
ERBB2 status				0,040			0,854
negative	95	58 (77%)	37 (60%)		50 (70%)	45 (68%)	
positive	42	17 (23%)	25 (40%)		21 (30%)	21 (32%)	
Pathological complete response, pCR				0,647			0,065
no	59	32 (65%)	27 (71%)		35 (78%)	24 (57%)	
yes	28	17 (35%)	11 (29%)		10 (22%)	18 (43%)	
5-year MFS (% [95CI])	121	45% [33-61]	53% [40-70]	0,437	47% [35-62]	53% [41-70]	0,273

Supplementary Table S4: Dataset of MMP2 and MMP9 expression levels (accession numbers in the ArrayExpress database: E-MTAB-1006 and E-MTAB-1547; GSE22597 in the GEO database)

See Supplementary File 1
