# Supplementary material for: MMP2 and MMP9 serum levels are associated with favorable outcome in patients with inflammatory breast cancer treated with bevacizumab-based neoadjuvant chemotherapy in the BEVERLY-2 study
Source: Oncotarget. 2016 Feb 23;7(14):18531–40. doi: 10.18632/oncotarget.7612 (PMC4951307; doi:10.18632/oncotarget.7612)
Supplement: Supplementary file 2 [file oncotarget-07-18531-s002.docx]

Supplementary table 4 - Dataset of MMP2 and MMP9 expression levels (accession numbers in the ArrayExpress database: E-MTAB-1006 and E-MTAB-1547; GSE22597 in the GEO database).

| SAMPLE_ID | DATASET_ID | Type | MMP2 (PID:201069_at) | MMP9 (PID:203936_s_at) |
| --- | --- | --- | --- | --- |
| TCRU_2425 | E-MTAB-1006 | IBC | 1,064 | 3,894 |
| TCRU_2437 | E-MTAB-1006 | IBC | 1,016 | 1,666 |
| TCRU_2441 | E-MTAB-1006 | IBC | 1,106 | 1,607 |
| TCRU_2442 | E-MTAB-1006 | IBC | -0,637 | 1,469 |
| TCRU_2443 | E-MTAB-1006 | IBC | 1,808 | 2,452 |
| TCRU_2444 | E-MTAB-1006 | IBC | -1,388 | 2,105 |
| TCRU_2445 | E-MTAB-1006 | IBC | -1,496 | 0,201 |
| TCRU_2446 | E-MTAB-1006 | IBC | 0,519 | 3,144 |
| TCRU_2449 | E-MTAB-1006 | IBC | 0,611 | 1,593 |
| TCRU_2458 | E-MTAB-1006 | IBC | 0,740 | 1,555 |
| TCRU_2459 | E-MTAB-1006 | IBC | 0,368 | 4,736 |
| TCRU_2542 | E-MTAB-1006 | IBC | 0,939 | 3,583 |
| TCRU_2545 | E-MTAB-1006 | IBC | 0,606 | -0,145 |
| TCRU_2613 | E-MTAB-1006 | IBC | 0,865 | 5,193 |
| TCRU_2614 | E-MTAB-1006 | IBC | 1,110 | 2,351 |
| TCRU_2615 | E-MTAB-1006 | IBC | 1,333 | 2,094 |
| TCRU_2619 | E-MTAB-1006 | IBC | -0,035 | 2,386 |
| TCRU_2620 | E-MTAB-1006 | IBC | 1,142 | 3,777 |
| TCRU_6388 | E-MTAB-1006 | IBC | -0,738 | 3,326 |
| TCRU_6389 | E-MTAB-1006 | IBC | -2,759 | 1,418 |
| TCRU_6390 | E-MTAB-1006 | IBC | -0,660 | 3,909 |
| TCRU_6391 | E-MTAB-1006 | IBC | 0,379 | 4,198 |
| TCRU_6392 | E-MTAB-1006 | IBC | 0,144 | 2,253 |
| TCRU_6393 | E-MTAB-1006 | IBC | 0,946 | 4,780 |
| TCRU_6394 | E-MTAB-1006 | IBC | 0,924 | 1,364 |
| TCRU_6395 | E-MTAB-1006 | IBC | -2,145 | 1,846 |
| TCRU_6396 | E-MTAB-1006 | IBC | 0,089 | -0,207 |
| TCRU_6397 | E-MTAB-1006 | IBC | 0,536 | -0,016 |
| TCRU_7891 | E-MTAB-1006 | IBC | -1,158 | 5,821 |
| TCRU_8409 | E-MTAB-1006 | IBC | -1,821 | 4,830 |
| TCRU_8410 | E-MTAB-1006 | IBC | 0,198 | 3,726 |
| TCRU_8411 | E-MTAB-1006 | IBC | 0,019 | 3,396 |
| TCRU_8412 | E-MTAB-1006 | IBC | -0,210 | 3,929 |
| TCRU_8413 | E-MTAB-1006 | IBC | -0,710 | 0,644 |
| TCRU_8414 | E-MTAB-1006 | IBC | 0,743 | 4,252 |
| TCRU_8415 | E-MTAB-1006 | IBC | -1,080 | -0,794 |
| TCRU_8416 | E-MTAB-1006 | IBC | 0,240 | 1,469 |
| TCRU_8417 | E-MTAB-1006 | IBC | -0,341 | 4,630 |
| TCRU_8418 | E-MTAB-1006 | IBC | -0,021 | 4,062 |
| TCRU_8419 | E-MTAB-1006 | IBC | -0,650 | 4,754 |
| TCRU_8420 | E-MTAB-1006 | IBC | -0,226 | 3,771 |
| IPC_IBC1 | E-MTAB-1547 | IBC | -0,297 | 0,396 |
| IPC_IBC2 | E-MTAB-1547 | IBC | -0,159 | 1,763 |
| IPC_IBC3 | E-MTAB-1547 | IBC | 0,305 | 2,900 |
| IPC_IBC4 | E-MTAB-1547 | IBC | 0,299 | 2,263 |
| IPC_IBC5 | E-MTAB-1547 | IBC | 0,172 | 4,695 |
| IPC_IBC6 | E-MTAB-1547 | IBC | -0,280 | 0,241 |
| IPC_IBC7 | E-MTAB-1547 | IBC | -0,757 | 3,416 |
| IPC_IBC8 | E-MTAB-1547 | IBC | -1,352 | 2,749 |
| IPC_IBC9 | E-MTAB-1547 | IBC | -3,535 | -0,277 |
| IPC_IBC10 | E-MTAB-1547 | IBC | 0,756 | 2,766 |
| IPC_IBC11 | E-MTAB-1547 | IBC | -0,256 | 1,586 |
| IPC_IBC12 | E-MTAB-1547 | IBC | 1,516 | -0,463 |
| IPC_IBC13 | E-MTAB-1547 | IBC | -2,657 | -0,464 |
| IPC_IBC14 | E-MTAB-1547 | IBC | -1,314 | 2,598 |
| IPC_IBC15 | E-MTAB-1547 | IBC | 0,662 | 1,090 |
| IPC_IBC16 | E-MTAB-1547 | IBC | -0,471 | 5,699 |
| IPC_IBC17 | E-MTAB-1547 | IBC | 0,519 | 0,757 |
| IPC_IBC18 | E-MTAB-1547 | IBC | 0,414 | 0,257 |
| IPC_IBC19 | E-MTAB-1547 | IBC | -0,193 | 1,796 |
| IPC_IBC20 | E-MTAB-1547 | IBC | -0,159 | 3,661 |
| IPC_IBC21 | E-MTAB-1547 | IBC | 0,869 | 1,171 |
| IPC_IBC22 | E-MTAB-1547 | IBC | -4,174 | 3,008 |
| IPC_IBC23 | E-MTAB-1547 | IBC | -0,098 | 1,903 |
| IPC_IBC24 | E-MTAB-1547 | IBC | 0,679 | 0,344 |
| IPC_IBC25 | E-MTAB-1547 | IBC | -0,420 | 2,332 |
| IPC_IBC26 | E-MTAB-1547 | IBC | -0,765 | 1,220 |
| IPC_IBC27 | E-MTAB-1547 | IBC | 0,803 | 2,758 |
| IPC_IBC28 | E-MTAB-1547 | IBC | 0,375 | 3,638 |
| IPC_IBC29 | E-MTAB-1547 | IBC | 1,310 | -0,450 |
| IPC_IBC30 | E-MTAB-1547 | IBC | 0,393 | 3,008 |
| IPC_IBC31 | E-MTAB-1547 | IBC | 0,201 | 1,156 |
| IPC_IBC32 | E-MTAB-1547 | IBC | -0,295 | 1,945 |
| IPC_IBC33 | E-MTAB-1547 | IBC | 0,484 | 1,027 |
| IPC_IBC34 | E-MTAB-1547 | IBC | 0,232 | 1,289 |
| IPC_IBC35 | E-MTAB-1547 | IBC | 0,886 | 0,910 |
| IPC_IBC36 | E-MTAB-1547 | IBC | 0,381 | 4,810 |
| IPC_IBC37 | E-MTAB-1547 | IBC | -0,480 | 0,878 |
| IPC_IBC38 | E-MTAB-1547 | IBC | -2,123 | -0,308 |
| IPC_IBC39 | E-MTAB-1547 | IBC | -0,035 | -0,006 |
| IPC_IBC40 | E-MTAB-1547 | IBC | 0,348 | 3,007 |
| IPC_IBC41 | E-MTAB-1547 | IBC | -0,261 | 1,549 |
| IPC_IBC42 | E-MTAB-1547 | IBC | -0,583 | 1,139 |
| IPC_IBC43 | E-MTAB-1547 | IBC | 0,206 | 2,881 |
| IPC_IBC44 | E-MTAB-1547 | IBC | 1,007 | 3,913 |
| IPC_IBC45 | E-MTAB-1547 | IBC | 0,830 | 4,674 |
| IPC_IBC46 | E-MTAB-1547 | IBC | -0,435 | 1,009 |
| IPC_IBC47 | E-MTAB-1547 | IBC | 0,450 | 1,217 |
| IPC_IBC48 | E-MTAB-1547 | IBC | 0,132 | 2,047 |
| IPC_IBC49 | E-MTAB-1547 | IBC | 1,239 | 2,669 |
| IPC_IBC50 | E-MTAB-1547 | IBC | -0,905 | -0,200 |
| IPC_IBC51 | E-MTAB-1547 | IBC | -2,365 | 4,224 |
| IPC_IBC52 | E-MTAB-1547 | IBC | -0,035 | 2,320 |
| IPC_IBC53 | E-MTAB-1547 | IBC | -0,647 | -1,716 |
| IPC_IBC54 | E-MTAB-1547 | IBC | 0,773 | 2,850 |
| IPC_IBC55 | E-MTAB-1547 | IBC | -1,889 | 2,741 |
| IPC_IBC56 | E-MTAB-1547 | IBC | 1,176 | 2,048 |
| IPC_IBC57 | E-MTAB-1547 | IBC | -1,751 | 0,919 |
| IPC_IBC58 | E-MTAB-1547 | IBC | -0,565 | 0,101 |
| IPC_IBC59 | E-MTAB-1547 | IBC | -3,476 | 2,542 |
| IPC_IBC60 | E-MTAB-1547 | IBC | -1,478 | 3,257 |
| IPC_IBC61 | E-MTAB-1547 | IBC | 0,256 | 1,156 |
| IPC_IBC62 | E-MTAB-1547 | IBC | 0,797 | 3,586 |
| IPC_IBC63 | E-MTAB-1547 | IBC | 1,447 | 4,188 |
| IPC_IBC64 | E-MTAB-1547 | IBC | -0,163 | 2,814 |
| IPC_IBC65 | E-MTAB-1547 | IBC | -1,981 | 3,592 |
| IPC_IBC66 | E-MTAB-1547 | IBC | -0,912 | 0,754 |
| IPC_IBC67 | E-MTAB-1547 | IBC | -1,306 | 1,177 |
| IPC_IBC68 | E-MTAB-1547 | IBC | -1,069 | 1,117 |
| IPC_IBC69 | E-MTAB-1547 | IBC | 0,246 | 0,419 |
| IPC_IBC70 | E-MTAB-1547 | IBC | 1,463 | 3,916 |
| IPC_IBC71 | E-MTAB-1547 | IBC | 1,283 | 2,305 |
| MDA_M116 | GSE22597 | IBC | -1,141 | -0,586 |
| MDA_M154 | GSE22597 | IBC | 0,001 | 3,641 |
| MDA_M230 | GSE22597 | IBC | -0,270 | 3,941 |
| MDA_M252 | GSE22597 | IBC | -0,311 | 2,077 |
| MDA_M279 | GSE22597 | IBC | -0,139 | 4,086 |
| MDA_M287 | GSE22597 | IBC | 0,233 | 0,965 |
| MDA_M292 | GSE22597 | IBC | 0,978 | 1,503 |
| MDA_M318 | GSE22597 | IBC | 0,075 | 5,451 |
| MDA_M347 | GSE22597 | IBC | 0,126 | 1,409 |
| MDA_M371 | GSE22597 | IBC | 0,545 | 1,112 |
| MDA_M402 | GSE22597 | IBC | -0,772 | 2,577 |
| MDA_M499 | GSE22597 | IBC | -0,656 | 2,226 |
| MDA_M514 | GSE22597 | IBC | -0,335 | 0,732 |
| MDA_M518 | GSE22597 | IBC | -0,923 | 0,573 |
| MDA_M526 | GSE22597 | IBC | -0,670 | 0,649 |
| MDA_M531 | GSE22597 | IBC | 3,242 | -0,102 |
| MDA_M534 | GSE22597 | IBC | -1,636 | 4,064 |
| MDA_M558 | GSE22597 | IBC | 2,075 | 1,817 |
| MDA_M572 | GSE22597 | IBC | -0,255 | 3,206 |
| MDA_M574 | GSE22597 | IBC | -0,979 | 1,357 |
| MDA_M603 | GSE22597 | IBC | -0,609 | 0,493 |
| MDA_M640 | GSE22597 | IBC | -0,694 | 0,469 |
| MDA_M642 | GSE22597 | IBC | -0,649 | 0,054 |
| MDA_M454 | GSE22597 | IBC | 0,082 | 7,126 |
| MDA_35606 | GSE22597 | IBC | -1,964 | 3,377 |
